# The economic effects of tax structure transformation: A study based on the tax reform of China's hainan free trade port

**DOI:** 10.1016/j.heliyon.2024.e39915

**Published:** 2024-10-29

**Authors:** Zuomin Zhang, Xun Xiao, Chunfeng Liu

**Affiliations:** a School of Economics, Hainan University, 570228, Haikou, China; b School of Economics, Yunnan University, 650091, Kunming, China; c School of Mathematics and Statistics, Guizhou University of Finance and Economics, 550025, Guiyang, China

**Keywords:** Tax structure, Sales tax, Hainan FTP, Computable general equilibrium

## Abstract

Many developing countries use tax reform as a strategy to support national or regional development. China's tax reform of the Hainan Free Trade Port in 2025 serves this purpose. The reform replaces the existing VAT, consumption tax, vehicle purchase tax, urban maintenance and construction tax, and education fee ("four taxes and one fee") with a sales tax. Previous studies of tax structure reform have rarely addressed the economic impact of such a transition, and few sales tax studies have discussed the issue of optimal tax rates. To study the economic effects of the introduction of sales tax and to find the optimal tax rate, this paper uses the computable general equilibrium (CGE) model to conduct simulation analyses. It is found that the sales tax rate should be 17.16 % to keep the fiscal revenue of Hainan Province more or less unchanged after the ‘four taxes and one fee’ are simplified into sales tax. The total economic volume and sales tax rate show an inverted ‘U’ type relationship, the optimal sales tax rate is about 9.79 %, and the tax rate of GDP, investment, employment growth of 3.82 %, 0.35 %, 3.41 %, respectively, the manufacturing industry and other industries in most of the different degrees of growth. Therefore, we suggest that the average sales tax rate of Hainan Free Trade Port should be around 9.79 %.

## 1 Introduction

It is common for governments to achieve policy goals by restructuring the tax system. In developed countries, aggressive tax system reforms rarely occur, and they usually achieve policy objectives through adjustments to a particular tax or tax rate. For example, in 2005 and 2006, Australia promoted national prosperity by reducing personal income taxes [1]. In the 1990s, some European countries (including Germany, the U.K., the Netherlands, and Denmark) introduced environmental taxes to encourage polluters to develop clean technologies [2]. In the Trump administration, the tax cut aimed to stimulate the economy [3]. Many developing countries usually perform tax reforms as a national or regional development strategy, hoping to achieve economic growth or promote fiscal performance. For example, in 1994, the tax-sharing reform in China included the intention to increase the central government's share of tax revenue [4]. After 2003, the replacement of sales tax with VAT in India aimed at eliminating "unhealthy" tax competition among states [5]. In the early 21st century, tax reforms in Chile and Argentina aimed to increase fiscal revenues and thus enhance the government's ability to reduce social income disparities [6]. The VAT reform in Burkina Faso aimed to increase government revenues and boost government investment [7]. To increase China's level of openness to the outside world and promote regional economic development, the Chinese government plans to build a free trade port in Hainan Province and implement aggressive tax reform in 2025 to make Hainan Province a highly developed free trade port like Hong Kong and Singapore. The reform will replace the current value-added tax, consumption tax, vehicle purchase tax, urban maintenance and construction tax, and education fee ("four taxes and one fee") with a sales tax, which will be levied on goods and services at the retail stage. The replacement of the current "four taxes and one fee" with a sales tax in Hainan Province starting in 2025 could be described as a huge social experiment. China has no historical experience in implementing sales tax and many issues need to be studied urgently. What are the possible effects of the aggressive tax reform? Does it have a promoting effect on local economic development? What is the optimal rate of sales tax? These issues have not been studied in the previous literature.

The study concerns two types of literature, the first of which relates to sales tax. Among the countries currently imposing sales tax, the U.S. was early in introducing it. In early 1932, in response to the financial pressure brought by the Great Depression, Mississippi first introduced a sales tax. By 1970, sales tax had been implemented in most U.S. states and became an important revenue source for local governments [8]. At present, many articles focus on the study of the United States sales tax, and some scholars have studied the structure of the United States sales tax system. Mikesell et al. believe that the current sales tax is a ‘narrow tax base, high tax rate’ tax structure, which is prone to distortions, inefficiencies, inequities, and difficulties in collection. It is also suggested that the most important way to minimize the problems created by the sales tax is to abandon taxing individual purchases of tangible property and focus on taxing household consumption expenditures [9]. Stunda argues that with a sales tax, the tax burden is shifted more equally between individuals and corporations than with an income tax and that corporations pay more taxes [10]. Some scholars study the prerequisites for implementing sales tax from the perspective of tax competition. Agrawal establishes a competitive model that includes different levels of local governments to analyze the interplay of sales tax rate settings among local governments at all levels. The results show a positive correlation between the sales tax rate between adjacent towns and a negative correlation between higher- and lower-level governments [11]. More scholars discuss the impact of the implementation of sales tax policy on employment, enterprise decision-making, and commodity prices. Burnes et al. analyze the relationship between sales tax rate and the employment of retail and manufacturing industries using data from Florida from 1992 to 2006 and find that one percentage increase in the local sales tax rate at the county level will increase the number of retail jobs by 258 and the number of manufacturing jobs by 838 [12]. Rohlin and Thompson use county-level quarterly data and a newly developed local county-level tax rate dataset to estimate the impact of sales taxes on state boundary employment. The results show that the increase in the sales tax rate harms employment, wages, and recruitment in border areas [13]. Bruce and Luna study how state sales tax policies affect the decisions of companies to establish business ties in each state. They emphasize that higher state sales tax rates and broader tax ranges reduce the tendency of e-commerce companies to establish taxable businesses in states with those policies [14]. Brock et al. discuss the impact of the Minnesota cigarette sales tax on retail prices of tobacco and find that an increase in the sales tax rate on cigarettes will increase the average retail price of cigarette exceeding the sales tax rate on tobacco itself, indicating that the increase in the cigarette sales tax rate will be excessively transferred to consumers [15]. None of the above studies on sales tax address how the optimal sales tax rate should be determined.

Another category of relevant literature is related to tax structure transformation, which focuses on the effects on government revenue, residential income, and industrial structure. Among the effects on government revenue, Fox et al. studied the impact of shifting the statutory incidence of taxation from consumers to online and out-of-state retailers based on state-level panel data. Research has found that changing the statutory incidence of taxation can reduce tax evasion, increase tax revenue, and improve economic efficiency [16]. Daba and Mishra analyze Ethiopia's tax structure reform using tax data between 1974 and 2013 and find that the tax reform needed to increase total tax revenue or shift the tax structure from indirect to direct taxes [17]. Using unbalanced panel data of 95 developing countries over the period 1981–2015, Gnangnon and Brun empirically analyze the impact of reforms that gradually replace trade taxes with domestic taxes on tax performance and show that implementing appropriate tax reforms in the context of greater trade openness will generate higher tax revenues [18]. Concerning the impact on residential income, Xu et al. investigate the impact of the abolishment of agricultural taxes in China on farmers' income by using the data from the household survey and find that farmers' net income increases significantly after the tax reform [19]. Using a computable general equilibrium (CGE) model, Amir et al. assess the impact of income tax reform in Indonesia on key macroeconomic variables and find that under the balanced budget assumption, the reduction in personal and corporate income taxes promotes the economic growth, while leads to a reduction in poverty incidence [20]. Regarding the impact on industry structure, Meng uses a CGE model to simulate the impact of carbon tax reform on Australian agricultural sectors and finds that all agricultural sectors are negatively impacted to varying degrees [21]. Beckman et al. also use a CGE model to simulate the impact of the Tax Cuts and Jobs Act (TCJA) in 2008 on American agricultural sectors and find that the reform reduced the output of agricultural sectors [22]. Using a system dynamics model, Ojha and Vrat simulate the impact of the reform of goods and services tax in India and find that effective implementation of the reform accelerates the growth of the manufacturing industry [23].

Since the Hainan Free Trade Port (FTP) was proposed by the Chinese government in 2018 and will be officially established in 2025, when the FTP system and the new tax system start to take effect, no literature studies the possible economic effect of the tax structure transformation.

The difficulty in studying the economic effects of the tax structure transformation of the Hainan FTP is that the tax structure has yet to change, i.e., there is not enough observational data to study the economic effects by empirical regression methods. CGE models are able to describe the interlocking relationships between sectors of the national economy, and between accounts, and to simulate and predict the impact of policy changes on these relationships. So, following Mamboundou, Beckman et al., Fogarty and Jakeman, we use a CGE model to study the possible economic effects of the tax structure transformation [7,22,24]. Nevertheless, the CGE model needs to be improved because the open economy CGE model cannot be directly applied to study the Hainan FTP economy. Because Hainan FTP is a province of China, its relationship with mainland China is different from that of other countries with mainland China.

When using the CGE model to study the economic problems of Hainan FTA, the industrial linkages between other provinces in mainland China and Hainan Province should be treated as external linkages, i.e., imports and exports of industries in Hainan province. However, the Chinese input-output data already have the traditionally defined imports and exports of Hainan Province, which are the industrial linkages between Hainan Province and other countries outside China. To use the Chinese input-output data and at the same time to distinguish the industrial linkages of Hainan Province with other provinces in mainland China from the imports and exports of Hainan Province, we label the industrial linkages of Hainan Province with other provinces in mainland China by the inflow or outflow of goods and services when calibrating the parameters of the CGE model. This approach allows the CGE model to study a country's regional issues.

Under the CGE framework, we simulate three tax structure transformation scenarios for the Hainan FTA. The first one is a scenario where the sales tax rate leaves government revenue unchanged, the second one is a scenario where the sales tax rate maximizes the regional output, and the third one is a scenario where differentiated sales tax rates are imposed on different industries. Simulation results show that to maintain the current government revenue of Hainan province, the sales tax rate should be as high as 17.16 % after the "four taxes and one fee" are replaced by a sales tax, making GDP, investment, and employment increase by 3.4 %, 0.05 %, 2.99 %, respectively, but residential consumption decreases by 12.26 %. There is an inverted U-shaped relationship between the total output of the Hainan FTP and the sales tax rate, with an optimal sales tax rate of 9.79 %, which makes GDP increase by 3.82 % but makes government revenue suffer a loss of 11.2 %. The optimal tax rate varies from industry to industry. For most industries, the optimal tax rate is between 9 % and 10 %, and it is beneficial to have sales taxes at different rates for different industries.

Our research contributions are reflected in the following. First, it enriches the literature on the transformation of the tax structure, as the tax reform of the Hainan FTP provides us with just the right opportunity to study the possible economic effects of the simplification of 10.13039/100014797VAT, consumption tax, vehicle purchase tax, urban maintenance and construction tax, and education fee ("four taxes and one fee") into a sales tax. Even though there is some study on the transformation of sales tax or consumption tax into VAT [5,25], previous literature has yet to study the transformation of VAT or consumption tax into a sales tax. Second, we study the optimal tax rate when implementing a sales tax from the perspective of maximizing the total output. Clarke studies sales tax rates from a government revenue maximization perspective [26], Okawa and Iguchi [27], Zhang and Choi [28] study sales tax rates from a welfare maximization perspective, while no literature studies the optimal sales tax rate from the perspective of maximizing the GDP.

The remaining article is structured as follows. Section 2 introduces the background of the tax structure reform of Hainan FTP. Section 3 establishes the CGE model, which is suitable for studying regional issues. Section 4 presents Data Sources and calibrates the parameters of the CGE model. Section 5 provides and discusses the simulation results. Section 6 operates the robustness analysis. Section 7 concludes.

## 2 Background of tax structure transformation of the Hainan FTP

The Chinese government has chosen Hainan Province several times as a pilot area for economic reform. The first time was in 1988 when the Chinese government upgraded Hainan Island, which had previously been part of Guangdong Province, to a provincial administrative region and established a special economic zone with broader autonomy to develop the economy and make regulations than other provinces in mainland China. The second time was in 2008, when the Chinese government set up Hainan Province as an international tourism island which is a pilot area for reform and innovation of China's tourism industry, with the hope of building the island to be a world-class leisure and holiday destination. The third time was in 2018 when it was chosen to establish the Hainan FTP, a pilot zone that includes reform of the tax structure. Hainan Province has been repeatedly selected as a pilot reform zone, mainly because it is an island isolated from mainland China by the Qiongzhou Strait, with a small economy and population, accounting for only 0.7 % and 0.6 % of China (excluding Hong Kong, Macau, and Taiwan) in terms of population and economy output in 2021,^1^ respectively.

Chinese President Xi Jinping announced that the central government supported Hainan Province in establishing the Hainan FTP in a speech in 2018 for the 30th anniversary of establishing the Hainan special economic zone. The decision to establish the Hainan FTP, including the tax transformation in Hainan Province, was made under a complex background. From the international perspective, economic globalization and global governance consensus are challenged by unilateralism and trade protectionism, which makes the prospect of international trade and investment less optimistic, and cross-border supply chains at risk of disruption. From China's domestic perspective, China is in a critical period of transforming its development mode and promoting its economic structure. However, transitioning from high-growth mode to high-quality development mode faces enormous challenges. The establishment of an FTP, including the transformation of the tax system in Hainan Province, is not only a demonstration by the Chinese government to the world of China's determination to continue to open up but also is an exploration of an alternative mode of economic development, with a hope of accumulating useful experience that can be applied to the whole country for higher-level reform.

The transformation of the tax structure is parallel with the construction of the Hainan FTP. The Twenty-ninth Session of the Standing Committee of the Thirteenth National People's Congress Passed the Law of the People's Republic of China on the Hainan Free Trade Port (Hainan FTP Law) on June 10, 2021. Article 27 of the Hainan FTP Law provides that: "Under the principles of simple and scientific tax structure, full optimization of the elements of the tax system, obvious reduction in the level of the tax burden, clear attribution of revenue, and basic balance of fiscal revenue and expenditure, the tax system of the Hainan FTP shall be established in line with the direction of national tax reform. When the whole island is closed for the operation of FTP, value-added tax, consumption tax, vehicle purchase tax, urban maintenance and construction tax, and education fee will be simplified and merged, and a sales tax will be levied on goods and services at the retail stage. After the whole island is closed for the operation of the Hainan FTP, the tax system will be further simplified." Currently, the urban maintenance and construction tax, and education fee in Hainan Province are taxes on taxes, i.e., the maintenance and construction tax, and education fee are based on the actual consumption tax and VAT paid. Therefore, when dealing with maintenance and construction tax, and education fee in the CGE model later, we can treat them as an additional tax based on consumption tax and VAT.

According to the Hainan FTP Law, the sales tax will replace the "four taxes and one fee" in 2025. Therefore, there is currently no data available about the tax structure transformation of the Hainan FTP that can be used to study. Following Fogarty and Jakeman [24], Beckman et al. [22], and Mamboundou [7], we use a CGE model to simulate the possible economic effects of the transformation of the tax structure of Hainan FTP. In addition, according to the Hainan FTP Law, after the "four taxes and one fee" are replaced with a sales tax, the sales tax should make the government keep a fiscal balance. Hence, we will simulate a scenario where the sales tax makes the government maintain a balanced fiscal status and calculate the sales tax rate, regional output, resident income, resident consumption, investment, employment, and social capital stock in this scenario.

## 3 Theoretical model

The introduction of a sales tax will have a wide-ranging impact on Hainan's macroeconomy and various industries, and an empirical assessment of this impact can provide a basis for the design of the sales tax. The CGE model is a powerful tool for analyzing and evaluating tax policy because it can describe the interlocking relationships between the various sectors of the national economy and the various accounts, and it can simulate and predict the impacts of policy changes on these relationships. To analyze the economic impact of the sales tax on the Hainan FTP from a general equilibrium perspective, we use a CGE model to simulate the impact of the sales tax on the Hainan FTP's macroeconomy and various industries after the tax structure transformation. The model is divided into six modules: production module, trade module, resident module, firm module, government module, and equilibrium and macro closure module. The model assumes that an industry produces only one good; the set of all industries is A, and the set of goods is C.

### 3.1 Production module

The production module consists of total output, intermediate, labor, and capital inputs. In this study, we use a two-layer nested production structure. In the first layer, the total output consists of intermediate inputs and synthetic factor inputs, and the production function takes a constant elasticity of substitution (CES) form as follows:

(1) $${QA}_{a}=\alpha_{a}{\left[\delta_{a}{QVA}_{a}^{\rho_{a}}+\left(1-\delta_{a}\right){QINTA}_{a}^{\rho_{a}}\right]}^{\frac{1}{\rho_{a}}}$$

(2) $$\frac{{PVA}_{a}}{{PINTA}_{a}}=\frac{\delta_{a}}{\left(1-\delta_{a}\right)}{\left(\frac{{QINTA}_{a}}{{QVA}_{a}}\right)}^{1-\rho_{a}}$$

(3) $${PA}_{a}\times {QA}_{a}=\left(1+{tbus}_{a}+{salt}_{a}\right)\left[{PVA}_{a}\times {QVA}_{a}+{PINTA}_{a}\times {QINTA}_{a}\right]$$

Equations (1), (2), (3) portray the total output, the ratio of added value price to intermediate input price, and the value of industry a, respectively. ${salt}_{a}$ denotes the combined rate of the consumption tax, vehicle purchase tax, urban maintenance and construction tax, and education fee. ${tbus}_{a}$ denotes all of the indirect tax rates other than the "four taxes and one fee". $\alpha_{a}$ denotes the scale parameter; $\delta_{a}$ denotes the share parameter; $\rho_{a}$ denotes the power parameter of the CES function, which is related to the elasticity of substitution; ${QA}_{a}$ denotes the output of industry a; ${QVA}_{a}$ denotes the added value of industry a; ${QINTA}_{a}$ denotes the reproduction input of industry a; ${PA}_{a}$ denotes the output price of industry a; ${PVA}_{a}$ denotes the price of the added value of industry a; and ${PINTA}_{a}$ denotes the price of the reproduction input of industry a.

The second-level production function has two parts: added value and intermediate input. The added value part is compounded by labor and capital through the CES function as follows:

(4) $${QVA}_{a}=\alpha_{av}{\left[\delta_{La}{QLD}_{a}^{\rho_{av}}+\left(1-\delta_{La}\right){QKD}_{a}^{\rho_{av}}\right]}^{\frac{1}{\rho_{av}}}$$

(5) $$\frac{WL}{WK}=\frac{\delta_{La}}{\left(1-\delta_{La}\right)}{\left(\frac{{QKD}_{a}}{{QLD}_{a}}\right)}^{1-\rho_{av}}$$

(6) $${PVA}_{a}\times {QVA}_{a}=\left(1+{vat}_{a}\right)\times \left(WK\times {QKD}_{a}+WL\times {QLD}_{a}\right)$$

Equations (4), (5), (6) portray the added value, the ratio of labor price to capital price, and the sum of the added value of industry a, respectively. ${QKD}_{a}$ denotes the demand for capital in industry a; ${QLD}_{a}$ denotes the demand for labor in industry a; WK denotes the price of capital; WL denotes the price of labor; $\rho_{av}$ denotes the power parameter of the CES function, which is related to the elasticity of substitution; ${vat}_{a}$ denotes the combined tax rate of VAT, urban maintenance and construction tax, and education fee. ${vat}_{a}$ should also be adjusted to zero after the sales tax is implemented.

Among them, the intermediate input is obtained through the Leontief composite, and the demand and price of intermediate inputs are calculated by Equations (7), (8), respectively.

(7) $${QINT}_{c\;a}={ica}_{c\;a}\times {QINTA}_{a}$$

(8) $${PINTA}_{a}=\sum_{c\in C}{ica}_{c\;a}\times {PQ}_{c}$$

where ${QINT}_{c\;a}$ represents the demand of industry a for product c, ${QINTA}_{a}$ refers to the intermediate input of industry a, ${ica}_{c\;a}$ is the direct consumption coefficient of input and output, ${PINTA}_{a}$ represents the total price of intermediate input, and ${PQ}_{c}$ represents the price of commodity c in the provincial market.

### 3.2 Regional trade module

In this study, we design a two-level nested constant transformation elasticity (CET) function to describe the industrial linkage between the Hainan FTP and other places. The first level of nesting is used to represent the industrial linkages between the Hainan FTP and foreign countries, i.e., imports and exports, and the second level of nesting is used to represent the industrial linkages between the Hainan FTP and provinces in mainland China,

(9) $${QA}_{a}=\alpha_{at}{\left[\delta_{at}{QDA}_{a}^{\rho_{at}}+\left(1-\delta_{at}\right){QE}_{a}^{\rho_{at}}\right]}^{\frac{1}{\rho_{at}}}$$

(10) $$\frac{{PDA}_{a}}{{PE}_{a}}=\frac{\delta_{at}}{\left(1-\delta_{at}\right)}{\left(\frac{{QE}_{a}}{{QDA}_{a}}\right)}^{1-\rho_{at}}$$

(11) $${PA}_{a}\times {QA}_{a}={PDA}_{a}\times {QDA}_{a}+{PE}_{a}\times {QE}_{a}$$

(12) $${PE}_{a}={pwe}_{a}\times EXR$$

Equations (9), (10), (11), (12) form the first part of the first-level nesting. Equation (9) shows that goods produced in the Hainan FTP (${QA}_{a}$) are used for domestic sales (${QDA}_{a}$) and exports (${QE}_{a}$). Equation (10) shows the ratio of the price of goods produced in the Hainan FTP and sold in other provinces in mainland China (${PDA}_{a}$) to the price of exported goods (${PE}_{a}$). Equation (11) shows the sum of the value of goods produced in the Hainan FTP. Equation (12) shows that the price of exported goods is affected by the exchange rate (EXR); ${pwe}_{a}$ is the price of goods expressed in foreign currency; $\rho_{at}$ denotes the power parameter of the CET function, which is related to the elasticity of substitution.

(13) $${QDA}_{a}=\alpha_{ap}{\left[\delta_{ap}{QPA}_{ap}^{\rho_{at}}+\left(1-\delta_{ap}\right){QPE}_{ap}^{\rho_{at}}\right]}^{\frac{1}{\rho_{at}}}$$

(14) $$\frac{{PPA}_{ap}}{{PPE}_{ap}}=\frac{\delta_{ap}}{\left(1-\delta_{ap}\right)}{\left(\frac{{QPE}_{ap}}{{QPA}_{ap}}\right)}^{1-\rho_{at}}$$

(15) $${PDA}_{a}\times {QDA}_{a}={PPA}_{ap}\times {QPA}_{ap}+{PPE}_{ap}\times {QPE}_{ap}$$

Equations (13), (14), (15) form the first part of the second-level nesting, representing the industrial linkages between the Hainan FTP and other provinces in mainland China. Equation (13) shows that the goods produced in the Hainan FTP and sold to domestic market (${\text{QDA}}_{a}$) go to the market of the Hainan FTP (${QPA}_{ap}$) and outflow to other provinces in mainland China (${QPE}_{ap}$). Equation (14) shows the ratio of the price of goods produced and sold in the Hainan FTP (${PPA}_{ap}$) to the price of goods produced in the Hainan FTP and outflow to other domestic provinces (${PPE}_{ap}$). Equation (15) shows the sum of the value of goods produced and sold in the Hainan FTP.

The goods sold in the market in the Hainan FTP consist of imported goods, goods produced and sold in the Hainan FTP, and an inflow of goods from other provinces in mainland China.

(16) $${QQ}_{c}=\alpha_{c}{\left[\delta_{c}{QDC}_{c}^{\rho_{c}}+\left(1-\delta_{c}\right){QM}_{c}^{\rho_{c}}\right]}^{\frac{1}{\rho_{c}}}$$

(17) $$\frac{{PDC}_{c}}{{PM}_{c}}=\frac{\delta_{c}}{\left(1-\delta_{c}\right)}{\left(\frac{{QM}_{c}}{{QDC}_{c}}\right)}^{1-\rho_{c}}$$

(18) $${PQ}_{c}\times {QQ}_{c}={PDC}_{c}\times {QDC}_{c}+{PM}_{c}\times {QM}_{c}$$

(19) $${PM}_{c}={pwm}_{c}\times EXR$$

Equations (16), (17), (18), (19) form the second part of the first-level nesting. Equation (16) shows that the goods sold in the market in the Hainan FTP (${QQ}_{c}$) are composed of goods domestically produced (${QDC}_{c}$) and imported goods (${QM}_{c}$). Equation (17) shows the ratio of the price of goods domestically produced (${PDC}_{c}$) to the price of imported goods (${PM}_{c}$). Equation (18) shows the sum of the value of goods sold in the market in the Hainan FTP. Equation (19) shows that the price of imported goods is affected by the exchange rate (EXR); ${pwm}_{c}$ is the price of imported goods expressed in foreign currency; $\rho_{c}$ denotes the power parameter of the Armington conditions, which is related to the elasticity of substitution.

(20) $${QDC}_{c}=\alpha_{cp}{\left[\delta_{cp}{QPC}_{cp}^{\rho_{c}}+\left(1-\delta_{cp}\right){QPM}_{cp}^{\rho_{c}}\right]}^{\frac{1}{\rho_{c}}}$$

(21) $$\frac{{PPC}_{cp}}{{PPM}_{cp}}=\frac{\delta_{cp}}{\left(1-\delta_{cp}\right)}{\left(\frac{{QPM}_{cp}}{{QPC}_{cp}}\right)}^{1-\rho_{c}}$$

(22) $${PDC}_{c}\times {QDC}_{c}={PPC}_{cp}\times {QPC}_{cp}+{PPM}_{cp}\times {QPM}_{cp}$$

Equations (20), (21), (22) form the second part of the second-level nesting. Equation (20) shows that goods domestically produced and sold in the Hainan FTP (${QDC}_{c}$) are composed of goods produced in the Hainan FTP (${QPC}_{cp}$) and the inflow from other domestic provinces (${QPM}_{cp}$). Equation (21) shows the ratio of the price of goods produced in the Hainan FTP (${PPC}_{cp}$) to the price of inflow from other domestic provinces (${PPM}_{cp}$). Equation (22) represents the total value of goods domestically produced and sold in the Hainan FTP. ${PPM}_{cp}$ is a price without "four taxes and one fee". The "four taxes and one fee" are levied on the inflow of goods from other domestic provinces, which are reflected in Equations (3), (6). After the introduction of the sales tax, ${salt}_{a}$ and ${vat}_{a}$ in Equations (3), (6) will be adjusted to zero because the Article 29 of the Hainan FTP Law mentions that “goods entering Hainan Free Trade Port from the Mainland shall be refunded the VAT and consumption tax that are levied under the relevant provisions of the State Council.”^2^

(23) $${QPC}_{cp}=\sum_{a}{IDENT}_{ac}\times {QPA}_{ap}$$

(24) $${PPC}_{cp}=\sum_{a}{IDENT}_{ac}{\times PPA}_{ap}$$

Where ${IDENT}_{ac}$ is the unit matrix, ${QPA}_{ap}$ and ${QPC}_{cp}$ are the goods produced and sold in the Hainan FTP, and ${PPA}_{ap}$ and ${PPC}_{cp}$ are the prices of goods produced and sold in the Hainan FTP. Equation (23) establish the one-to-one correspondence between ${QPA}_{ap}$ and ${QPC}_{cp}$, and Equation (24) establish the one-to-one correspondence between ${PPA}_{ap}$ and ${PPC}_{cp}$.

### 3.3 Resident module

The resident module contains two parts: residents' income and expenditure. Residents' income comes from labor, capital, and government transfers to residents. The relationship among these is shown in Equation (25). Residents’ expenditure is composed of consumption, savings, and personal income taxes paid to the government. The sales tax is levied at the retail stage of goods and services, and the goods and services sold at the retail stage are consumed by residents and the government. The model is designed to tax the goods and services consumed by residents and the government at the retail stage. Referring to Bhattarai et al. [29] on the design of the CGE model of retail sales tax, we make the sales tax levied on residents and government:

(25) $$YH=WL\times QLS+{shif}_{hk}\times WK\times QKS+{transfr}_{hg}$$

(26) $${PQ}_{c}\times \left(1+{tsal}_{c}\right)\times {QH}_{c}={shrh}_{c}\times mpc\times \left(1-{ti}_{h}\right)YH$$

where Y.H. denotes residents' income, ${shif}_{h\;k}$ denotes the share of total capital income, QLS denotes labor supply, QKS denotes capital supply, ${transfr}_{h\;g}$ denotes government transfer payment to residents, ${QH}_{c}$ denotes residents' consumption of goods, ${shrh}_{c}$ denotes residents' consumption of goods c as a share of total consumption, mpc denotes residents' average propensity to consume, ${ti}_{h}$ denotes the personal income tax rate, and ${tsal}_{c}$ denotes the sales tax rate after the tax is levied.

### 3.4 Enterprise module

The income of enterprises comes from the income obtained from the invested capital and transfer payment from the government to enterprises. The savings of enterprises equal the income of enterprises minus the income tax. The total investment equals the sum of each sector's investment. The specific relationship is shown in Equations (27), (28), (29). In the following equations, YENT denotes the income of enterprises, ${shif}_{ent\;k}$ denotes the proportion of capital income of enterprises to total capital income, ${transfr}_{ent\;g}$ denotes the transfer payment from the government to enterprises, ENTSAV denotes the savings of enterprises, ${ti}_{ent}$ denotes the corporate income tax rate, EINV denotes total investment, and ${QINV}_{c}$ denotes investment of sector c.

(27) $$YENT={shif}_{entk}\times WK\times QKS+{transfr}_{entg}$$

(28) $$ENTSAV=\left(1-{ti}_{ent}\right)YENT$$

(29) $$EINV=\sum_{c}{PQ}_{c}\times {QINV}_{c}$$

### 3.5 Government module

The government module contains two parts: government revenue and government expenditure. The revenue of local governments in the Hainan FTP comes from the central government's transfer payment and the tax revenue, where tax revenue is comprised of sales tax, other indirect taxes, personal income tax, and corporate income tax. Government expenditure is used for government consumption of goods and transfers to enterprises and residents. The difference between revenue and expenditure is credited to government savings. The specific relationship is shown in the equations below:

(30) $$TSA=\sum_{c}{tsal}_{c}\times {PQ}_{c}\times \left({QH}_{c}+{QG}_{c}\right)$$

(31) $$TBS=\sum_{a}\frac{{tbus}_{a}+{salt}_{a}}{1+{tbus}_{a}+{salt}_{a}}{PA}_{a}\times {QA}_{a}+\sum_{a}{vat}_{a}\times \left(WK\times {QKD}_{a}+WL\times {QLD}_{a}\right)$$

(32) $$YG=TBS+TSA+{ti}_{h}\times YH+{ti}_{ent}\times YENT+{transfr}_{govg}$$

(33) $$EG=\sum_{c}{PQ}_{c}\times \left(1+{tsal}_{c}\right)\times {QG}_{c}+{transfr}_{hg}+{transfr}_{entg}$$

(34) $${PQ}_{c}\times \left(1+{tsal}_{c}\right)\times {QG}_{c}={shrg}_{c}\left(EG-{transfr}_{hg}-{transfr}_{entg}\right)$$

(35) GSAV=YG−EG

After transforming "four taxes and one fee" into a sales tax, the sales tax is reflected in Equation (30), where ${tsal}_{c}$ denotes the sales tax rate. The newly introduced sales tax is levied at the retail stage of goods and services, so ${tsal}_{c}$ is the tax rate levied on government and residential consumption. Before the transformation of the tax structure, ${salt}_{a}$, and ${vat}_{a}$ are not equal to zero, while ${tsal}_{c}$ is equal to zero. After the transformation of the tax structure, ${salt}_{a}$, and ${vat}_{a}$ became zero, while ${tsal}_{c}$ becomes positive. In the above equations, TSA denotes sales tax revenue of the Hainan FTP, TBS denotes indirect tax revenue of the Hainan FTP other than that derived from "four taxes and one fee", Y.G. denotes government revenue of the Hainan FTP, E.G. denotes government expenditure of the Hainan FTP, ${transfr}_{gov\;g}$ denotes transfer payment from central government to the Hainan FTP's government, ${QG}_{c}$ denotes the government consumption of goods, ${shrg}_{c}$ denotes the share of government consumption on commodity c in total consumption, and GSAV denotes government savings.

### 3.6 Equilibrium and macro closure module

The equilibrium of the model includes the equilibrium of the commodity market, the equilibrium of the factor market, and the equilibrium of the trade market. In the commodity market, the total supply equals the total demand, composed of intermediate demand, residential demand, investment demand, and government demand, as shown in Equation (36). In the labor market, the total demand for labor in the factor market equals the total supply, as shown in Equation (37). In the capital market, the total demand for capital equals the total supply, as shown in Equation (38). In the domestic and foreign trade market, the difference between imports and exports is the foreign savings FSAV, as shown in Equation (39). In the trade market between the Hainan FTP and provinces in mainland China, the central government's transfer payment (${transfr}_{govg}$) is considered as part of inflow. Denoting the outside province saving by PROSAV, we can describe the market equilibrium between the Hainan FTP and other provinces by Equation (40). In terms of investment savings, total investment equals total savings, as shown in Equation (41). Considering the fact that China has a large number of surplus labor. This paper establishes macro closure with Keynesian closure characteristics. Labor prices, capital prices, exchange rates, government savings, and exogenous provincial savings are exogenous.

(36) $${QQ}_{c}=\sum_{a}{QINT}_{c\;a}+{QH}_{c}+{QINV}_{c}+{QG}_{c}$$

(37) $$\sum_{a}{QLD}_{a}=QLS$$

(38) $$\sum_{a}{QKD}_{a}=QKS$$

(39) $$\sum_{c}{pwm}_{c}\times {QM}_{c}=\sum_{a}{pwe}_{a}\times {QE}_{a}+FSAV$$

(40) $${PPM}_{cp}\times {QPM}_{cp}={PPE}_{ap}\times {QPE}_{ap}+{transfr}_{govg}+PROSAV$$

(41) $$EINV=\left(1-{mpc}_{h}\right)\left(1-{ti}_{h}\right)\times YH+ENTSAV+GSAV+EXR\times FSAV+PROSAV$$

## 4 Data Sources and parameter calibration

### 4.1 Data

The social accounting matrix (SAM) is the database of the CGE model. SAM is prepared based on an input-output table. Because of the complexity of the input–output table, the preparation of the table requires a lot of human and material resources, which makes the Chinese government provides the table every five years ending with 2 or 7. We provide the Hainan SAM based on the Hainan input–output table in 2017.^3^ The data on intermediate inputs, consumption by residents and the government, investment, imports and exports, and domestic and extra-provincial inflows and outflows are mainly derived from input-output tables. Other data are from the China Taxation Yearbook 2018 and the Hainan Statistical Yearbook 2018.

We adjust the industries into 19 sectors according to the current industrial status and available data. Industry and corresponding codes are shown in appendix A. According to the research needs, we also set up labor, capital, resident, enterprise, government, sales tax, VAT, indirect tax, other indirect tax, personal income tax, corporate income tax, investment, out-of-province accounts.^4^,^5^,^6^

Data that are from different sources, collected by different statistical calibers, or missing will make SAM inaccurate and unbalanced between rows and columns. Because the input-output table provides adequate data and is balanced between rows and columns, the data from the input-output table prevail when data are available from different sources. Some goods flowing from Hainan Province to other provinces in China are imported through Hainan Customs, but these goods do not enter the production processes in Hainan Province and flow out of the province directly. They are not deducted from the outflow in the statistics, leading to negative numbers in the production and sales in Hainan Province, which violate the economic theory. Therefore, we adjust the inflow and outflow of the province in the input–output table.

### 4.2 Parameter calibration

The parameters involved in the model can be calibrated with SAM except for the elasticity coefficients. The elasticity of substitution in the CES function, the elasticity of substitution in the CET function, and the elasticity of substitution in the Armington condition are derived from the GATP database. The power parameter ρ can be derived by ρ=1−1/ε, where ε is the elasticity of substitution of the function. When the power parameter ρ is known, the corresponding scale parameter and share parameter can be obtained by combining SAM.

The direct consumption coefficient is calculated through the intermediate input part. The share of residents' consumption of a certain type of commodity to total consumption (${shrh}_{c}$) and the share of government consumption of a certain commodity to total consumption (${shrg}_{c}$) are calculated through residents’ and government consumption in the SAM table.

## 5 Simulation analysis

According to the Hainan FTP Law, the tax structure transformation should maintain a fiscal balance of the Hainan FTP government. Therefore, we study the economic effects in a scenario where the tax structure transformation keeps government revenue unchanged. In addition, we also want to find out whether there are sales tax rates that will produce better economic effects. Therefore, in the second scenario, we study the economic effects of various sales tax rates. Because one kind of tax often imposes different tax rates on different industries, we study the third scenario, which involves imposing differentiated tax rates on different industries.

### 5.1 Sales tax rate that keeps government revenue unchanged

In this scenario, the "four taxes and one fee" are replaced with a sales tax, and the government revenue remains unchanged.^7^ For the convenience of calculation and discussion, we assume that the sales tax rate is the same for all industries. After simulation, we find that the sales tax rate (${tsal}_{c}$) should be as high as 17.2 %. This rate is higher than the current basic VAT rate (13 %). The reason is that the sales tax is levied only at the retail stage of goods and services, while the current VAT is levied at all stages of production and services and therefore has a broad tax base. At the same time, sales tax includes the current consumption tax, vehicle purchase tax, urban maintenance and construction tax, and education fee.

Compared to other literature that studies sales tax in the Hainan FTP, the sales tax rate (17.16 %) simulated in this article is high. For example, Zhang and He propose that the sales tax rate should be 5 % [30]. There are two reasons for the difference. The first reason is that the objectives of the study are different. The objective of the sales tax in this article is to keep the government of the Hainan FTP maintain a fiscal balance after the government replaces the "four taxes and one fee" with the sales tax, while the objective of the sales tax in the study by Zhang and He [30] is to promote the service industries, such as tourism industry. The second reason is that the tax bases are different. This article selects the consumption expenditure of residents and the government as the tax base to avoid double taxation. In contrast, the study by Zhang and He [30] selects the sum of total retail sales of consumer goods, total sales of commercial housing, tourism income, postal and telecommunications business income, and express business income as the tax base of sales tax. The tax base chosen by Zhang and He [30] is much broader but will involve repeated collection, leading to the underestimation of the sales tax rate. Another study by Xu and Zhang [31], which has the same tax base as that in Zhang and He [30], proposes a sales tax of 13 % to compensate for the loss of the central government's tax revenue transfer due to replacement of the "four taxes and one fee" with a sales tax. Xu and Zhang also propose a sales tax of 15 % if the government of the Hainan FTP wants to keep a fiscal balance [31]. Because of a broader tax base, the tax rates in both cases are lower than the rate simulated in this article.

In countries and regions where the sales tax is levied, the tax rates are much lower than the tax rate calculated here. For example, 45 states in the U.S. currently have sales, and most states' sales taxes, combined with city and county sales taxes, are less than 10 %.^8^ In Canada, only the provinces of Saskatchewan, British Columbia, and Manitoba currently levy sales taxes, with sales tax rates of 6 %, 7 %, and 7 %, respectively.^9^ The difference stems mainly from differences in the tax structures. Direct taxes comprise a large proportion of government revenue in The U.S. and Canada, while indirect taxes comprise a large proportion of government revenue in Hainan Province. After the transformation of the tax structure, only by implementing a sales tax with a high rate can the government of the Hainan FTP maintain a fiscal balance.

Table 1 reports the changes in the main macroeconomic variables in the Hainan FTP when the sales tax rate is 17.16 %. The simulation results show that after “the four taxes and one fee” are replaced with the sales tax, the GDP will increase by 3.4 %, residents' income will increase by 2.8 %, residents’ consumption will decrease by 12.26 %, the total investment will increase by 0.05 %, social employment will increase by 2.99 %, and social capital stock will increase by 3.72 %. GDP increased after the tax reform, despite the high sales tax rate, because the tax burden on firms falls, increasing firms' willingness to invest. However, after the tax reform, residents' consumption declines significantly because the tax burden on residents increases significantly.

**Table 1:** Percentage changes in major macroeconomic variables.

Variable name	GDP	Government revenue	Resident income	Resident consumption	Total investment	Social employment	Social capital stock
Change in percentages	3.400	−0.003	2.798	−12.258	0.053	2.993	3.722

**Fig. 1:**
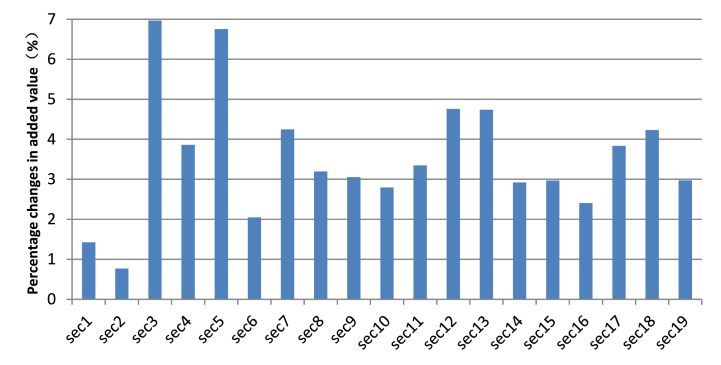
Percentage changes in the added value of each industry.

Fig. 1 simulates the change in the added value of each industry in the Hainan FTP after a sales tax with a rate of 17.16 % replaces the "four taxes and one fee". The results show that the added value of all industries increased. Except for the industries of agriculture, forestry, animal husbandry and fishery products and services, the added value of all industries increased by more than 2 percent. The most significant growth is seen in manufacturing and construction, which increased by 6.97%and 6.76 % respectively. Manufacturing and construction industries get the most benefit because most products in manufacturing and construction industries enter the process of production as intermediate goods. However, sales tax is levied only at the final consumption stage, making no sales tax levied on the goods in the manufacturing and construction industries. In addition, the added value of the industry in transportation, storage and postal services, the industry in leasing and business services, and the industry in scientific research and technical services has grown by more than 4 %. The products or services of the three industries are provided mainly to enterprises in the production process, so introducing a sales tax reduces the burden on the three industries, increasing their added value.

The industry in agriculture, forestry, animal husbandry and fishery products and services has a growth rate of 1.42 %. The goods and services of these sectors are mainly for residents' consumption at the final stage of consumption, and most of their products and services are the objects of the sales tax. At the same time, before the tax structure transformation, most of the products in the industry are exempt from VAT, with the lowest tax burden. The introduction of a sales tax increases the industry's tax burden, so the growth in the industry's added value is lower than that of most other industries.

### 5.2 Economic effects under different sales tax rates

We will explore whether there are tax rates that will produce better economic effects in this scenario. Since VAT revenue accounts for more than 70 % of the total revenue of the “four taxes and one fee”, the primary sales tax rate can be set in various scenarios by referring to the VAT rate. The primary VAT rate was reduced from 17 % to 16 % on May 1, 2018, and from 16 % to 13 % on April 1, 2019. In addition to the primary rate, there are preferential VAT rates of 9 % and 6 %. Therefore, we want to simulate the economic effects when the sales tax rate is 6 %, 9 %, 13 %, and 17 %, respectively. At the same time, in order to observe the relationship between the sales tax rate and the GDP, we set up two additional cases with sales tax rates of 9.79 % and 10 %, respectively.

Table 2 shows the changes in the main macroeconomic variables under different sales tax rates. When the sales tax rate is 6 %, the GDP increases by 3.49 %. The GDP gradually increases with the sales tax rate. When the tax rate is 9.79 %, the GDP reaches the maximum with an increase of 3.82 %. After that, further increases in the tax rate lead to a gradual decrease in the GDP, indicating an inverted U-shaped relationship between the total output of the Hainan FTP and the sales tax rate, with an optimal sales tax rate of 9.78 %. This is because the sales tax is levied in the final stage of consumption, increasing the prices of consumption goods and making residents consume less. The higher the sales tax rate is, the lower the proportion of total output for consumption and the higher the proportion for investment. When the sales tax rate is low, increasing the sales tax rate brings an increase in investment, thus increasing total social capital, which is conducive to improving the GDP. At this stage, investment plays a leading role in raising the GDP. However, when the sales tax rate is high, further increases in the sales tax rate have a disproportionately negative impact on residents' consumption and lead to a reduction in aggregate demand, which is detrimental to the economy. This inverted U-shaped relationship between tax rates and the GDP is consistent with the study by Bania et al. [32] and Liu [33].

**Table 2:** Percentage changes in macroeconomic variables under different sales tax rates.

Tax rate	GDP	Government revenue	Resident income	Resident consumption	Total investment	Social employment	Social capital stock
6.00 %	3.494	−17.139	2.820	−3.000	0.176	3.007	3.927
9.00 %	3.537	−12.513	2.890	−5.606	0.177	3.086	3.919
9.79 %	3.821	−11.197	3.184	−6.017	0.346	3.408	4.189
10.00 %	3.803	−10.880	3.173	−6.207	0.330	3.397	4.158
13.00 %	3.504	−6.360	2.886	−8.950	0.136	3.846	3.086
17.00 %	3.409	−0.245	2.805	−12.133	0.068	2.999	3.734

**Fig. 2:**
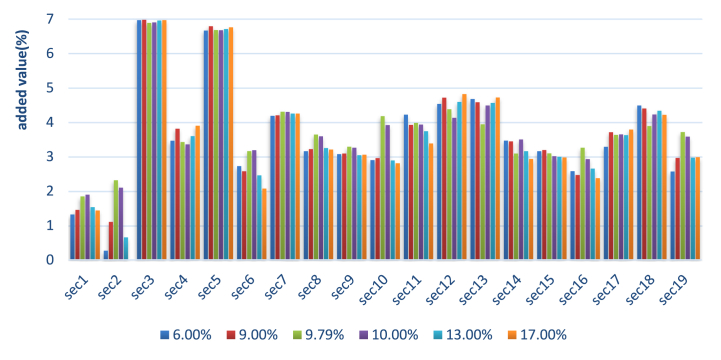
Percentage changes in the added value of each industry under various rates of sales tax.

Fig. 2 reports the percentage changes in industries' added value under various sales tax rates. The added value of most industries increases with the sales tax rate when the sales tax rate is low but decreases with the sales tax rate when the sales tax rate is high, evidence that there is an inverted U-shaped relationship between the sales tax rate and the added value of most industries. However, the optimal tax rate varies by industry. To be specific, when the sales tax rate is lower than 17 %, there is an inverted U-shaped relationship between the sales tax rate and the added value of the industry in agriculture, forestry, animal husbandry, and fishery products and services, the industry in mining, the industry in the wholesale and retail trade, the industry in the transportation, storage and postal services, the industry in accommodation and catering, the industry in information transmission, software and information technology services, the industry in finance, the industry in residential services, repair and other services, the industry in education, and the industry in real estate, with an optimal tax rate between 9 % and 10 %.

In summary, the introduction of a sales tax has a positive impact on the economy of the Hainan FTP. After the transformation of the tax structure, there is an inverted U-shaped relationship between the GDP of the Hainan FTP and the sales tax rate. The average optimal sales tax rate for the Hainan FTP is about 9.8 %. Under the optimal tax rate, the GDP, resident income, total investment, and social capital stock are all at their maximum. The optimal tax rate varies from industry to industry, so it may be beneficial to have sales taxes at different rates for different industries.

### 5.3 Economic effects of sales tax rate with industry differences

It is a common practice in countries with retail sales taxes to impose different tax rates on different industries. For example, Malaysia levies a sales tax of 10 % on taxable goods and 6 % on taxable services, and the sales tax rates are divided into two main classes.^10^ At the same time, the above analysis indicates that imposing different sales tax rates on different industries may increase the added value of some industries. In this subsection, we further investigate the economic effects of implementing different sales tax rates for different industries. Since VAT accounts for the major part of the revenue of the "four taxes and one fee”, when designing the sales tax rate for different industries, we can refer to the VAT rates. However, it violates the original purpose of simplifying the tax system by introducing a sales tax with too many rates. Therefore, we set one primary and two preferential tax rates when designing the sales tax rates. The specific tax schemes are as follows.

Scheme I: A zero rate is imposed on the industry in agriculture, forestry, animal husbandry and fishery products and services, and the primary rate of 17.16 % is imposed on other industries. Because agriculture-related products and services are exempted from VAT in China, it is reasonable to impose a sales tax with zero rates on agriculture-related industries.

Scheme II: A zero rate is imposed on the industry in agriculture, forestry, animal husbandry and fishery products and services. A primary rate of 17.16 % is imposed on industry in mining, industry in manufacturing, industry in electricity, heat, gas and water production and supply, industry in the wholesale and retail trade, industry in real estate, industry in leasing and business services, and industry in residential services, repair and other services. A preferential tax rate of 6 % is imposed on other industries because, before the transformation of the tax structure, these industries were enjoying a preferential VAT rate.

Scheme III: A preferential tax rate of 9 % is imposed on the industries which are taxed at 6 % in Scheme II, and the remaining industries are taxed at the same rates as in Scheme II.

Table 3 reports the changes in the main macroeconomic variables under three tax schemes. Under Scheme I, the GDP will increase by 3.52 %, gaining more than that in the first scenario where the agriculture-related industry is without the exemption of the sales tax, and the GDP increases by 3.4 %. However, the government of the Hainan FTP will suffer a mild fiscal imbalance under this tax scheme. Under Scheme II, the increment in the GDP is more than that under Scheme I, but the government of the Hainan FTP also faces more revenue loss. Under Scheme III, the increment in the GDP is even greater, but the government revenue loss is less than that of Scheme II. Comparing the three sales tax schemes, we can find that Scheme III is best from the perspective of the GDP. Because there are differences in the optimal tax rate of various industries, the optimal tax rate of some industries is lower than the primary sales tax rate (17.16 %). Thus, implementing lower sales tax rates for some industries will result in better growth in those industries and ultimately lead to better macroeconomic outcomes. However, the three schemes do not provide a distinct advantage to the overall economy compared to implementing a uniform optimal sales tax rate on all industries.

**Table 3:** Percentage changes in macroeconomic variables under various tax schemes.

Variable name	GDP	Government revenue	Resident income	Resident consumption	Total investment	Social employment	Social capital stock
Scheme I	3.517	−3.56	3.375	−9.446	−1.296	3.68	3.219
Scheme II	3.548	−12.369	2.921	−6.603	0.148	3.123	3.897
Scheme III	3.596	−9.958	2.956	−7.508	0.19	3.158	3.978

Table 4 shows the percentage changes in the added value of each industry when different sales tax rates are imposed on different industries. The added value of the agriculture-related industry, which includes agriculture, forestry, animal husbandry and fishery products and services, increases most under Scheme II, even though the average sales tax rate is highest in this tax scheme because the industry is exempted from the sales tax under this tax scheme. Compared to Scheme III, most of the industries under Scheme II experience varying degrees of decline in added value. The added value of most industries under Scheme II and III is higher than that under Scheme I. One of the reasons for this is that reductions in sales tax rates in some industries increase the consumption of residents, which will further drive the growth of all industries. Another reason for this is that growth in some industries increases the demand for other industries due to the interconnectedness of the industries. Thus, the GDP increases more when differentiated tax rates are imposed on different industries than when the same tax rate is imposed on all industries.

**Table 4:** Percentage changes in the added value of each industry under various tax schemes.

Industry	Scheme I	Scheme II	Scheme III
Agriculture, forestry, animal husbandry and fishery products and services	5.166	1.598	1.515
Mining	2.709	2.225	2.201
Manufacturing	4.443	6.855	6.904
Production and supply of electricity, heat, gas and water	1.076	3.011	3.929
Architecture	4.413	6.821	6.651
Wholesale and retail	4.308	2.776	2.612
Transportation, storage and postal services	4.060	4.304	4.315
Accommodation and catering	2.310	3.631	3.442
Information transmission, software and information technology services	2.034	3.094	3.252
Finance	0.459	2.908	3.324
Real estate	3.571	4.060	3.924
Leasing and business services	−1.553	3.461	4.374
Scientific research and technical services	3.889	4.446	4.147
Water conservancy, environment and public facilities management	3.227	1.963	3.234
Resident services, repairs and other services	4.325	2.257	3.024
Education	3.622	2.865	2.867
Health and social work	0.534	2.497	3.702
Culture, sports and entertainment	3.308	4.140	3.979
Public administration, social security and social organizations	−1.709	3.050	3.284

## 6 Robustness analysis

In the CGE model, all parameters except the elasticity coefficients can be calibrated from the SAM table, so the selection of the elasticity coefficients will have an impact on the results of the simulation. To ensure the reliability of the simulation results, we provide sensitivity analysis. Following Mamboundou [7] we increase and decrease the elasticity coefficients we use in the benchmark model by 50 %, respectively, and then simulate the model with a sales tax rate of 17.16 %. To compare the results based on new elasticity coefficients with those in Table 1, we report the results in Table 5. It shows that after a 50 % increase in the elasticity coefficients, the directions of changes in all macroeconomic variables, except for the total investment, are the same as those in Table 1, and after a 50 % decrease in the elasticity coefficients, the directions of changes in all macroeconomic variables are also the same as those in Table 1.

**Table 5:** Percentage changes in macroeconomic variables based on other elasticity coefficients.

	GDP	Government revenue	Resident income	Resident consumption	Total investment	Social employment	Social capital stock
50 % increase in the elasticity coefficients	1.8704	−0.7072	1.6489	−13.2392	−0.2523	1.7864	1.7786
50 % decrease in the elasticity coefficients	3.4466	−0.2441	2.8412	−12.2216	3.224	3.0375	3.7959

In addition, to observe the sensitivity of the percentage changes in the added value of each industry to elasticity coefficients, we simulate the model with a sales tax rate of 17.16 % for a 50 % increase and decrease in the elasticity coefficients, respectively. Table 6 shows the results of each industry's percentage changes in added value based on the new elasticity coefficients. It shows that after a 50 % increase in the elasticity coefficients, the directions of percentage changes in the added value of all industries, except those in finance and real estate, are the same as those in Fig. 1, Fig. 2. After a 50 % decrease in the elasticity coefficients, the directions of percentage changes in the added value of all industries are the same as those in Fig. 1.

**Table 6:** Percentage changes in the added value of each industry based on other elasticity coefficients.

Industry	50 % increase in the elasticity coefficients	50 % decrease in the elasticity coefficients
Agriculture, forestry, animal husbandry and fishery products and services	1.8381	0.5837
Mining	1.0052	2.2436
Manufacturing	6.0092	6.9731
Production and supply of electricity, heat, gas and water	1.7503	4.9898
Architecture	4.6486	6.9452
Wholesale and retail	0.6577	0.2992
Transportation, storage and postal services	2.2031	4.4862
Accommodation and catering	1.6146	5.9020
Information transmission, software and information technology services	1.5762	5.4098
Finance	1.0280	3.8541
Real estate	−1.2231	2.4565
Leasing and business services	1.9874	5.6017
Scientific research and technical services	3.6151	4.4542
Water conservancy, environment and public facilities management	2.5605	2.9861
Resident services, repairs and other services	2.3241	4.1655
Education	−4.0203	3.5880
Health and social work	2.7507	4.6686
Culture, sports and entertainment	3.4058	4.4946
Public administration, social security and social organizations	1.2312	5.0357

Meanwhile, the simulation results with different elasticity coefficients show that the industry with the most significant increase in the added value is manufacturing, the same as in the baseline simulation. The new simulation results also indicate a high sales tax rate of around 17 % to maintain a fiscal balance and show that introducing a sales tax will positively impact the macroeconomy and most industries. To some extent, the above results validate the simulation results' robustness. Overall, the above results validate the robustness of the basic conclusions of this article to a certain extent.

## 7 Conclusion, policy implications and limitation

### 7.1 Conclusion

Establishing an FTP and carrying out aggressive tax reform in Hainan Province is a huge social experiment rarely seen worldwide. There is no study on the economic effects of replacing the VAT with a sales tax and no study investigating the optimal rate for the sales tax from the perspective of maximizing total regional output.

We embed the inflow and outflow of goods and taxes into provincial regions in a CGE model, thus making it possible to study regional tax reform in a general equilibrium framework and then simulate the economic effects of the tax structure transformation in the context of the Hainan FTP. The results indicate that: (i) after the “four taxes and one fee” are replaced with a sales tax, the sales tax rate should be as high as 17.16 % to maintain the current revenue of Hainan Province. This tax rate is higher than that of Zhang and Xu's research, and the reason for this is due to different research objectives and selected tax bases [30,31]. Although the tax rate is relatively high, simulation results show that levying a sales tax at this rate will have a positive impact on the macroeconomy.The replacement of the “four taxes and one fee” with the sales tax at a rate of 17.16 % will make GDP, investment, and employment of the Hainan FTP increase by 3.4 %, 0.05 %, and 2.99 %, respectively. However, residential consumption will suffer a decrease of 12.26 % at the sales tax rate. At the same time, the added value of each industry will increase, especially for industries with a higher proportion of goods entering the production process as intermediate inputs. (ii) There is an inverted U-shaped relationship between the GDP of the Hainan FTP and the sales tax rate, with an optimal average sales tax rate of 9.79 %, which increases the total output by 3.82 %, but makes government revenue suffer a loss of 11.2 %. There is an inverted U-shaped relationship between the added value of most industries and the sales tax rate, but the optimal sales tax rate varies among industries. (iii) Imposing different sales tax rates on different industries is beneficial to the increase in the added value of most industries. However, it does not provide a distinct advantage to the overall economy compared to imposing a uniform optimal sales tax rate on all industries.

### 7.2 Policy implications

Based on the above findings, this paper makes the following recommendations:

First, the average sales tax rate of Hainan Free Trade Port should be around 9.79 %. Hainan's GDP and sales tax rate show an inverted ‘U’ type relationship, with the sales tax rate of 9.79 % when GDP increases the most. When the tax rate is higher than this value, the GDP growth rate starts to decline, and the negative effect brought by increasing the tax rate exceeds the positive effect, which is unfavorable to the improvement of the macroeconomic environment and the development of the industry. After the introduction of sales tax, no matter how its tax system is designed, the actual tax burden generated should be controlled at around 9.79 %.

The second strengthens the system design to prevent sales tax evasion. According to the simulation results of this paper, the sales tax rate to keep the government revenue of Hainan Province roughly unchanged should be 17.16 %, and if this is the goal of collecting sales tax, then the sales tax rate is high. The high tax rate will increase the probability of tax evasion, and at the same time, tax evasion will cause the erosion of the tax base, leading to the decline of the government's sales tax revenue. Therefore, when designing the sales tax system, the problem of tax evasion caused by high tax rates should be taken into account and the sales tax collection and management system should be improved as much as possible.

Thirdly, accelerate the pace of tax reform and look for new tax sources to make up for the loss of tax revenue brought by sales tax. According to the research of this paper, the optimal sales tax rate with the best economic effect is much lower than the sales tax rate that keeps the government revenue more or less unchanged, and the long-term construction of Hainan's free trade harbor will ultimately have to collect sales tax at the optimal rate. In order to keep government revenues from falling, Hainan should accelerate the pace of tax reform and find new tax sources to make up for the loss of tax revenues from the sales tax. For example, try to implement property tax, because Hainan Free Trade Port can be the first to try in economic system reform and social governance innovation, etc., and has the legal conditions for the introduction of property tax, which is conducive to the increase of government revenue in Hainan Province.

### 7.3 Limitation

The CGE model is a powerful tool for analyzing and evaluating the economic effects of policies, but its simulation accuracy will be affected when simulating and predicting the real economy due to the defects of model design and calculation. The accuracy and reliability of the CGE model are highly dependent on the quality and completeness of the input data, especially in the elasticity parameter values. According to the rigor of empirical research, the estimated values of the elasticity parameters selected in this paper should be subjected to econometric empirical tests, however, this work is a large amount of engineering, and due to the limited research time and energy, this paper basically refers to the relatively authoritative Purdue University's GTAP database for the selection of the values of these elasticity parameters, and the subsequent research can be dedicated to the estimation of the elasticity parameters in the model to carry out relevant econometric empirical tests, so that these parameters are more accurate and more reliable. test, so that these parameters have more real statistical significance and increase the prediction accuracy of the simulation results.

## CRediT authorship contribution statement

**Zuomin Zhang:** Writing – review & editing, Writing – original draft, Supervision, Resources, Project administration, Methodology, Investigation, Funding acquisition, Formal analysis, Conceptualization. **Xun Xiao:** Writing – review & editing, Writing – original draft, Validation, Resources, Methodology, Investigation, Formal analysis, Data curation. **Chunfeng Liu:** Visualization, Software, Data curation.

## Data availability

Data will be made available on request.

## Declaration of competing interest

The authors declare that they have no known competing financial interests or personal relationships that could have appeared to influence the work reported in this paper.

## Figures and Tables

**Table:** 

Industry code	Industry
sec1	Agriculture, forestry, animal husbandry and fishery products and services
sec2	Mining
sec3	Manufacturing
sec4	Production and supply of electricity, heat, gas and water
sec5	Architecture
sec6	Wholesale and retail
sec7	Transportation, storage and postal services
sec8	Accommodation and catering
sec9	Information transmission, software and information technology services
sec10	Finance
sec11	Real estate
sec12	Leasing and business services
sec13	Scientific research and technical services
sec14	Water conservancy, environment and public facilities management
sec15	Resident services, repairs and other services
sec16	Education
sec17	Health and social work
sec18	Culture, sports and entertainment
sec19	Public administration, social security and social organizations

## Footnotes

1 The data are from China's official statistics bureau.

2 Please refer to Article 29 of the law of the People's Republic of China on the Hainan Free Trade Port (Order No. 85 of the president of the People's Republic of China).

3 Data sources: National Economic Accounting Department of the National Bureau of Statistics. 2020. China Regional input-output Table. China Statistics Press: 250–261.

4 The VAT account includes the VAT, urban maintenance and construction taxes, and education fee levied based on the actual amount of VAT paid.

5 The indirect tax account includes the consumption tax, vehicle purchase tax, urban maintenance and construction tax, and education fee levied based on the actual amount of consumption tax paid.

6 Other indirect tax account includes indirect taxes other than those in the VAT and indirect tax accounts.

7 Referring to Article 27 of Hainan FTP Law.

8 Source: Sales Tax Institute, https://www.salestaxinstitute.com/resources/rates.

9 Source: Retail Council of Canada, https://www.retailcouncil.org/resources/quick-facts/sales-tax-rates-by-province/.

10 Source: Malaysia Customs, http://www.customs.gov.my/ms/Pages/sst.aspx
